# Characterization of nitrogen doped graphene bilayers synthesized by fast, low temperature microwave plasma-enhanced chemical vapour deposition

**DOI:** 10.1038/s41598-019-49900-9

**Published:** 2019-09-23

**Authors:** C. R. S. V. Boas, B. Focassio, E. Marinho, D. G. Larrude, M. C. Salvadori, C. Rocha Leão, D. J. dos Santos

**Affiliations:** 1 Federal University of ABC, Centro de Engenharia, Modelagem e Ciências Sociais Aplicadas, Santo André, 09210-580 Brazil; 2 University of Twente, Industrial Focus Group XUV Optics, MESA+ Institute for Nanotechnology, Enschede, 7522 NH The Netherlands; 3 Graphene and Nano-materials Research Center - MackGraphe, Mackenzie Presbyterian University, São Paulo, 01302-907 Brazil; 4 Physics Institute, University of São Paulo, São Paulo, 05508-090 Brazil

**Keywords:** Surfaces, interfaces and thin films, Mechanical and structural properties and devices

## Abstract

New techniques to manipulate the electronic properties of few layer 2D materials, unveiling new physical phenomena as well as possibilities for new device applications have brought renewed interest to these systems. Therefore, the quest for reproducible methods for the large scale synthesis, as well as the manipulation, characterization and deeper understanding of these structures is a very active field of research. We here report the production of nitrogen doped bilayer graphene in a fast single step (2.5 minutes), at reduced temperatures (760 °C) using microwave plasma-enhanced chemical vapor deposition (MW-PECVD). Raman spectroscopy confirmed that nitrogen-doped bilayer structures were produced by this method. XPS analysis showed that we achieved control of the concentration of nitrogen dopants incorporated into the final samples. We have performed state of the art parameter-free simulations to investigate the cause of an unexpected splitting of the XPS signal as the concentration of nitrogen defects increased. We show that this splitting is due to the formation of interlayer bonds mediated by nitrogen defects on the layers of the material. The occurrence of these bonds may result in very specific electronic and mechanical properties of the bilayer structures.

## Introduction

Since graphene was effectively isolated in 2004^1^, its outstanding properties, such as flexibility, mechanical resistance, high intrinsic mobility and electromechanical modulation^2–9^ have attracted huge interest from researchers. The absence of a band-gap, however, has prevented its implementation as a possible replacement in semiconductor industry^10^.

For monolayer graphene, both theoretical and experimental^11–14^ studies have demonstrated that an effective option towards its electronic modulation is nitrogen doping, which generates n-type extrinsic electrical conductivity^15^. The substitution of carbon by nitrogen disrupts the ideal sp^2^ hybridization of graphene’s lattice, locally inducing significant changes to its electronic properties, separating the conduction band from the valence band even at modest concentrations of 2%^11,16^. This possibility of tunning graphene’s electronic properities could lead to potential applications in electronic devices, from electrochemical biosensors to spintronics^17,18^.

Another possibility for implementing a band-gap in graphene is working with few layer graphene films. It has long been known that the application of an electric field can induce the appearance of a band-gap in bilayer graphene^10^. Last year, Y. Cao *et. al*. have shown that by twisting the relative angle between two graphene sheets the resulting electronic properties of the system can vary from a Mott-insulator^19^ to a superconductor^20^. Other groups have since then shown that twisting the angle between the layers of other 2D heterostructures - in what has been called twistronics - has also been achieved for materials such as MoSe_2_-WS_2_^21^ and even heterostructures composed of three layers of atomic sheets alternating 2D Boron Nitride sheets with graphene monolayer^22^. In all of these cases, the appearance of a new periodicity in the rotated crystalline systems - called a moiré superlattice - results in new electronic properties of the systems. These properties can be so drastically different and so sensitive to the twist angle that changes in the relative rotation of the layers by no more than 0.2 degrees can cause all the remarkable effects observed to disappear^20^. The effective combination of 2D materials into heterostructures already appeared as a technique that would unveil an enormous new set physical properties that could be employed for a vast set of possible technological applications. The realization that the properties of these systems can be so dramatically altered by twisting the angle of stacking these monolayers indicates that these new possibilities stretch even further^23^.

These exciting new findings have brought renewed interest to the study of bilayer grahene and methods to synthesize, dope and characterize these systems. Moreover, the comprehension of how atomic defects specific to bilayer systems affect their electronic and mechanical properties is of paramount importance. In this work we demonstrate a plasma-enhanced chemical vapor deposition (PECVD) process for the synthesis and doping of graphene bilayer structures in a single step. By carefully adjusting the variables of the plasma (e.g. power, operating pressure and process duration), and flow ratio of gases (nitrogen, hydrogen and methane) we were able to obtain nitrogen-doped bilayer sheets in one single fast process (2.5 minutes). Moreover, we were able to carry out this process at low temperature (760 °C). Through Raman spectroscopy we could verify the effectiveness of the synthesis, determining that we obtained large area graphene bilayer films. We also confirmed doping thought the appearance of D′ band, attributed to the defect-induced intravalley double resonance scattering process^24,25^. Another relevant feature observed was the shifting of 2D peaks to lower energies, related to the introduction of electron donors (such as nitrogen) into the samples^26–32^. Using XPS we confirmed the presence of nitrogen defects in the synthesized material, identifying that pyridine defects prevail in our samples. However, as the concentration of nitrogen in the bilayer graphene is increased, we observed in the high resolution N1s spectrum an unexpected splitting in the peak associated to these defects.

If the observed splitting in the XPS peak was associated to new types of defects becoming more frequent in the material as the concentration of nitrogen increases, we would expect similar behavior to occur in the monolayer graphene, but we have found no reports of a similar splitting in the literature for this material. Therefore, we hypothesized that this splitting could be caused by new conformations of the usual pyridinic defects exclusive to bilayer graphene, called clipping^33^. In this conformation, an interlayer covalent bond forms, linking defects on the two layers of the material. We used quantum-mechanical parameter-free simulations, based on the density functional theory, to test this hypothesis. We simulated several defect complexes involving nitrogen and carbon vacancies, including pyridine and pyrrolic in different orientations. We also investigated how these defects could interact with a second layer of graphene, both pristine or containing other defects, employing different levels of simulations, with and without the explicit inclusion of van der Waals interaction. The ideal relative proportions of these defects in the synthesized material is readily given by their formation energies. For some defect complexes, we observed that the formation of covalent bonds between nitrogen in one layer with one of the atoms in the layer adjacent to it is stable. We then performed calculations of level shifts associated to core electrons of nitrogen in different sets of defects, simulating the XPS signature of each of these defects complexes, comparing to our experimental data. We conclude that the splitting of the XPS peak in our bilayer graphene samples as the concentration of nitrogen increased is likely related to the formation of the interlayer bonds mediated by nitrogen defect complexes. The presence of these interlayer covalent bonds can affect the mechanical as well the electronic properties of bilayer graphene in significant contrast to the way these defects modulate the properties of monolayer graphene. Therefore, the induction or suppression of such defects can represent another parameter to be taken into consideration when tailoring graphene and other bilayer or few layer heterostructures for specific applications.

## Results and Discussion

### Experimental results and discussion

**Figure 1:**
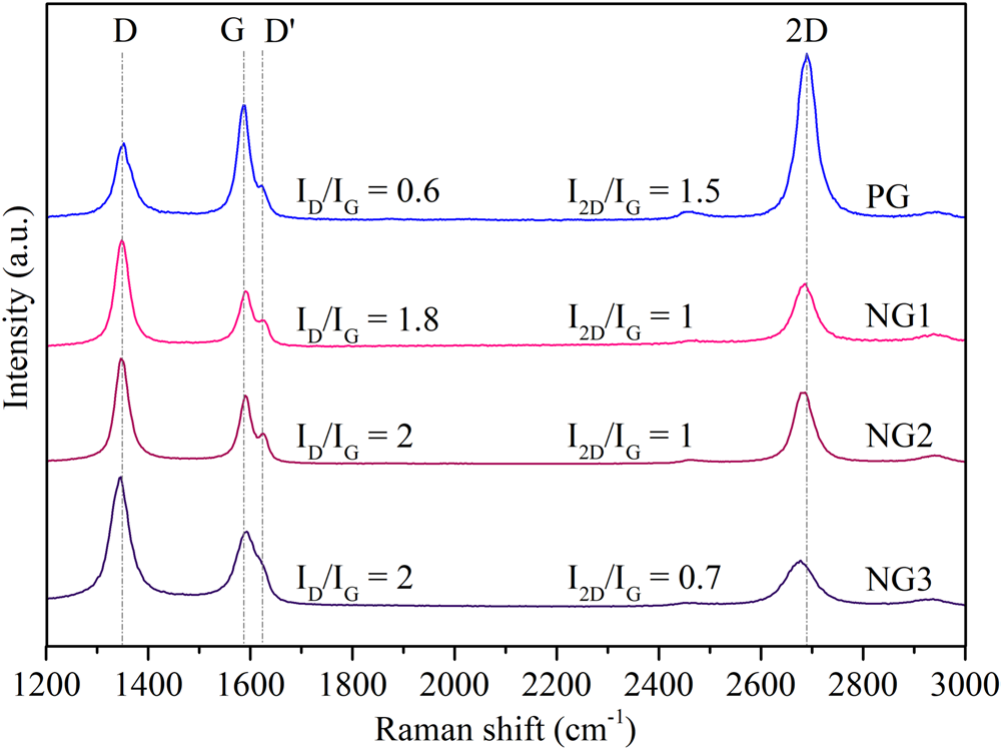
Raman spectra of pristine and nitrogen doped samples synthesized according to parameters depicted in Table 5.

We performed Raman analyses of our graphene samples to study their structural properties as well as dopant content. Figure 1 shows the Raman spectra of PECVD-graphene on SiO_2_/Si substrate for pristine (PG) and nitrogen-doped samples (NG1, NG2, NG3). The intensity, the shape and the peak position are strong indicatives of the number of layers, graphene structure and type of dopants^24,33–36^.

In the spectra of all samples, Fig. 1, the G band can be identified. This is a feature common to both graphene and graphite. We also note the 2D band, which can be used to distinguish graphene from graphite. In this context, the number of layers in a structure can be characterized by the relative intensities of these two peaks (I_2*D*_/I_*G*_) and by the full width at half-maximum (FWHM) of the 2D band^37^. A monolayer graphene usually presents I_2*D*_/I_*G*_ values of 2 or bigger with a FWHM ~30 cm^−1^, while bilayer structures present this intensity ratio ranging from around 1 to 1.5 and a FWHM ~50 cm^−1 ^^35,38–40^. Therefore, the structures synthesized in our experiments show typical intensity relation values of bilayer graphene. Raman mapping studies also confirm the high-yield growth of bilayer graphene as can be seen in Supplementary Information Fig. S1. Other techniques to assess the film thickness of the samples could be employed, such as the optical contrast and the ratio of Si 2p and C 1s signal of survey XPS spectrum. This would require, however, precise knowledge of the stoichiometry of the SiO_2_ substrate, as well as careful removal of carbon and other impurities left on the sample during the transfer to the substrate. Therefore, we believe that the Raman analyses described above is more indicated in our case.

Peaks at 1350 cm^−1^ (D band), and an additional peak located at ~1620 cm^−1^ (D′ peak) are also observed. The D and D′ peaks are activated by inter and intra-valley double resonant Raman process^24,25^, in which the defects are responsible for the missing momentum that completes the resonant process. Therefore, the increase in the concentration of defects leads to an increase in both intensities^41,42^. Pristine sample shows a moderate amount of defects (D band) even without nitrogen doping. This is expected as the growth occurs in the presence of hydrogen plasma, which would contain energetic ions and radicals that induce defect formation^43,44^.

The 2D peak, on the other hand, originates from two-phonon double resonant process, which does not need defects to satisfy the resonant condition. Its intensity is affected by the electron/hole scattering rate. The presence of nitrogen dopants in graphene would both create defects and introduce electron doping, which increases the electron scattering rate and, consequently, decreases the intensity of the 2D peak^36,45^. With the increase of nitrogen incorporated in the sample, the D band increase together with the 2D band decrease and broadening then indicates the formation of more disordered structures^1,26–32^, which is further confirmed by the increase on the *I*_*D*_/*I*_*G*_ ratio^39^. Moreover, it has been shown that the in-plane vibrational G band of graphene and bilayer graphene is blue-shifted for both electron and hole doping, due to the non-adiabatic removal of the Kohn anomaly at the Γ point. On the other hand, the 2D band is red-shifted for electron doping and blue-shifted for hole doping, due to the charge transfer induced modification of the equilibrium lattice parameter^24,46^.

In our analysis, we found that the 2D band red-shifts while the G band blue-shifts as the nitrogen content increase during the synthesis process. In Fig. 1, the red-shifted 2D peaks are found in 2687 cm^−1^, 2684 cm^−1^ and 2679 cm^−1^, while the blue-shifted G peaks in 1593 cm^−1^, 1594 cm^−1^ and 1595 cm^−1^ for NG1, NG2 and NG3 samples, respectively. Therefore, the value and evolution on peak intensities combined with position shift consolidate strong evidences of bilayer graphene formation, with nitrogen dopants concentration increase proportional to nitrogen flow during the PECVD process.

**Figure 2:**
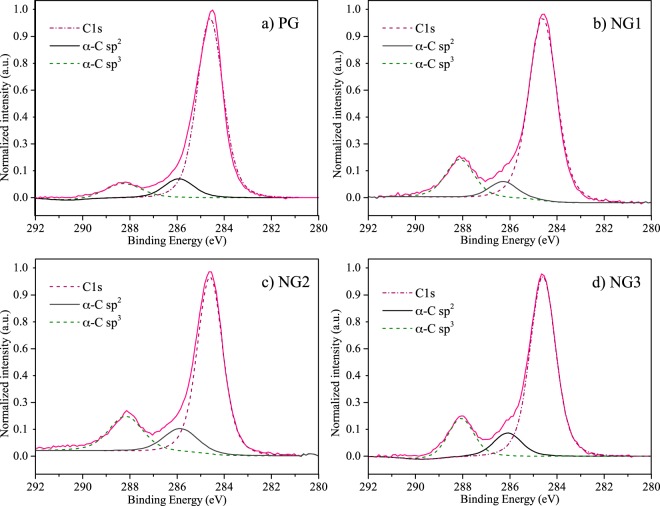
C1 s spectra of pristine and doped structures of graphene.

XPS was used to investigate chemical state changes on graphene structure as a result from nitrogen flow increasing during the synthesis, i.e., doping process. Fig. 2a–d depicts high resolution C spectrum for PG, NG1, NG2 and NG3, which revealed the presence of three main peaks in all samples. The C1s band, with highest intensity and centered around 284.6 eV, is associated with sp^2^ hybridized carbon and confirms the formation of graphene during the synthesis. A second and third bands, centered at 286 eV and 288.5 eV obtained by deconvolution of previously described peak, reflects the binding of both oxygen or nitrogen to sp^2^ C and sp^3^ C respectively^12,26,36^. Previous studies^17,42,47^ show that N-doped graphene is more susceptible for oxygen functionalization. This fact might be ascribed to the increased reactivity of atoms at the edge of defects and pyridinic N sites. These sites react with oxygen-containing species when exposed to the atmosphere, creating a sample with higher oxygen content. As our samples were exposed to laboratory air before the XPS analysis, the oxygen signature might be a consequence of the described phenomenon. The higher intensity at *α*-C sp^3^ position and of *α*-C sp^2^ band with the increase in nitrogen corroborates the observed Raman results, as these changes are caused by the increase of ligands concentration, which further confirms graphene doping.

**Figure 3:**
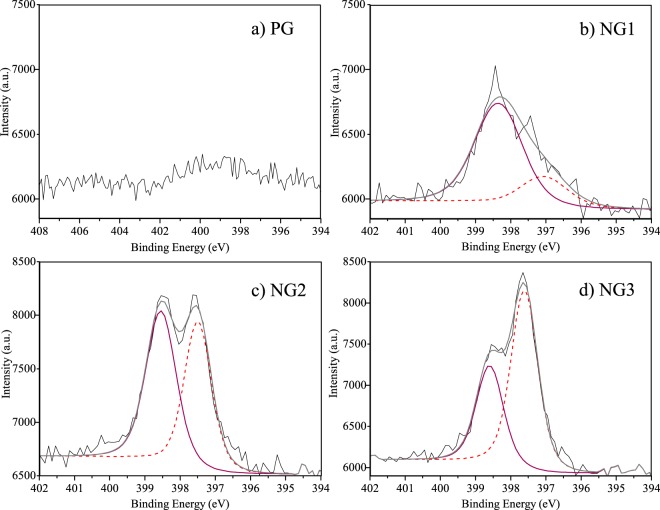
N1 s spectra of pristine and doped graphene structures.

Nevertheless, a proper comprehension of chemical state changes required the analysis of high resolution N spectra, which are presented in Fig. 3. Nitrogen binding in doped graphene is usually correlated with three common bonding configurations in XPS spectra: graphitic N (or substitutional N), pyrrolic N and pyridinic N, with binding energies respectively between the ranges 401.1–402.7 eV, 399.8–401.2 eV and 398.1–399.3 eV^12,13,15,48^. Figure 3a does not show nitrogen related peaks in the PG spectrum, confirming that the sample obtained in this synthesis condition is indeed pristine. On the other hand, Fig. 3b shows a high intensity peak at about 398.4 eV for NG1 sample, indicating the dominance of pyridine species in our bilayer graphene. The chemical composition of the samples (PG, NG1, NG2 and NG3) can be estimated from survey XPS spectra, curves similar to the ones shown in Fig. S2 for samples PG and NG3, available in Supplemental Information. Figure 3 is a magnification of such graphs. The peak areas, determined after background subtraction using the Shirley’s method, indicate nitrogen atomic percentages for the sample NG1, NG2, NG3 and PG of 2.0 at.%, 4.2 at.%, 4.2 at.% and below 0.2 at.%, respectively. The splitting of the single peak of sample NG1 into a peak slightly shifted to higher energies (~398.4 eV) and a second peak centered around 397.5 eV indicate the possibility of an increase in concentration of another type of nitrogen related defect, whose concentrations can be inferred by the same method. The area of this split-off peak increase from 0.5 at.%, to 2.0 at.% and to 2.7 at.% for the samples NG1, NG2 and NG3, respectively.

Previous studies have assigned this second peak in the XPS analysis to N–Cu bonding in Cu_3_N, in which the compound was fabricated by reactive radio frequency sputtering of copper targets in nitrogen and argon environment^49^. Nevertheless, it is known that copper nitrate is not stable at temperatures higher than 400 °C^12,49^. Therefore, the formation of such compound is inconsistent with our experimental conditions (760 °C temperature synthesis), which indicates that the observed peak might arise from other binding structures present in doped bilayer graphene. To further investigate the chemical state changes detected by our XPS measurements, we performed fully quantum atomistic simulations based on the density functional theory (DFT) method.

### Theoretical results and discussion

**Figure 4:**
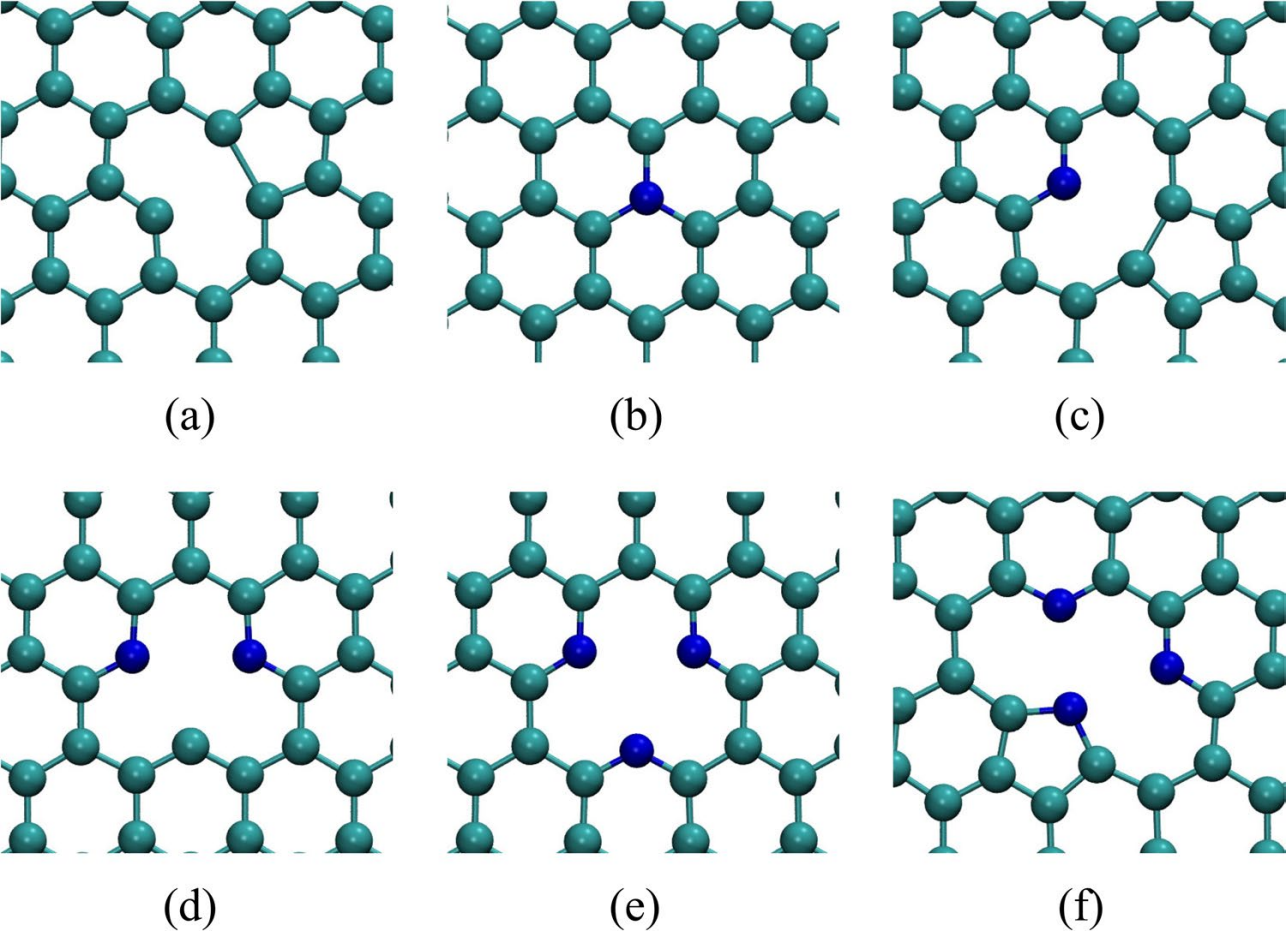
Relaxed geometry of defects in monolayer graphene. (**a**) Single vacancy (SV); (**b**) Substitutional nitrogen (subst.); (**c**) Monomerized pyridine (Mon. pyri.); (**d**) Dimerized pyridine (Dim. pyri.); (**e**) Trimerized pyridine (Trim. pyri.); (**f**) Trimerized pyrrole (Trim. pyrr.).

In Fig. 4 we show the relaxed geometry of the nitrogen defects in graphene simulated in this work. Our aim is to investigate these defects in graphene bilayer to try explain the origin of the splitting in the XPS curve discussed above.

We expect that coulomb interaction will favor the formation of complexes composed by defects of opposing charges, specially in bilayer graphene, in which defects of opposite charge on different layers can approach each other more easily than in the monolayer. In the Supplemental Information we discuss how we estimate the electronic state of the defects indicated in Fig. 4. As shown in Table S1 in Supplemental Information, substitutional and trimerized pyridine are negatively charged whereas vacancy, monomerized pyridine, dimerized pyridine and trimerized pyrrole are positively charged. The interaction between these defects will certainly cause structural distortion on each other. In some cases, this interaction could lead to the formation of interlayer covalent bonds mediated by the defects. As a matter of fact, even when defects are not attracted by electrostatic forces, the proximity of frustrated bonds in different layers can result in the formation of interlayer covalent bonds. Since XPS signal is strongly related to the chemical environment in which the atoms are located, such distortions could, in principle, result in XPS signals significantly different between the tabulated values associated to defects in the monolayer and the same defects in bilayer graphene. In Table S2 in Supplemental Information we show that the formation energy of the individual defects in the monolayer shown in Fig. 4 do not vary significantly in bilayer graphene when one of the layers is free from defects. We verified these results using optB86b + vdW-DF exchange and correlation functional^50–54^ which aims to accurately describe van der Waals forces in DFT simulations. The interlayer distances of AB and AA stackings considering vdW forces remained nearly the same as those obtained with LDA 3.34 Å and 3.54 Å. The binding changed markedly to −33 meV per atom, or three times more than predicted by LDA. We also simulated defects using optB86b + vdW-DF functional. In the Supplemental Information Fig. S4(a,b) we compare results using these two approaches for defect formation energy in monolayer and bilayer graphene, respectively. We observe almost no change in the formation energies of the defects in both cases using either optB86b + vdW-DF functional or LDA, being the variation for trimerized pyridin and pyrrole slightly more significant.

**Table 1:** Defect complex formation energy *E*_*f*_ using LDA, Δ*E*_*f*_ is the difference between the bilayer formation energy of the bilayer structure formed by two defects *E*_*f*_ and the formation energy of each defect in its monolayer graphene structure, $\Delta {E}_{f}={E}_{f,12}-({E}_{f\mathrm{,1}}+{E}_{f\mathrm{,2}})$.

Defect	Clipped?	*E*_*f*_^*LDA*^ (eV)	Δ*E*_*f*_^*LDA*^ (eV)
Mon. pyri. + SV	no	12.86	0.24
	yes	12.76	0.34
Mon. pyri. + subst.	no	4.70	0.50
Trim. pyri. + SV	no	10.33	0.36
	yes	9.81	0.52
Trim. pyrr. + SV	no	12.58	0.25
	yes	11.45	1.38
Trim. pyrr. + subst.	no	4.55	0.38
Trim. pyrr. + Trim. pyri.	no	7.16	0.52
	yes	7.68	0.46

**Table 2:** Calculated CLS for 1 s core electrons of monolayer and bilayer graphene defects. For a single vacancy, a C1s is used, all other results are related to N1s core eigenvalues.

Defect	*E*_*CLS*_ (eV)	
	Monolayer	Bilayer
Single Vacancy	121.8	126.7
Substitutional	0.0	1.5
Monomerized pyridine	3.0	4.6
Trimerized pyridine	3.1	4.7
Trimerized pyrrole	3.9	5.3

Where more than one nitrogen atom is present we computed the average value. Bilayer results obtained with the combination of a defective and a pristine layer.

**Figure 5:**
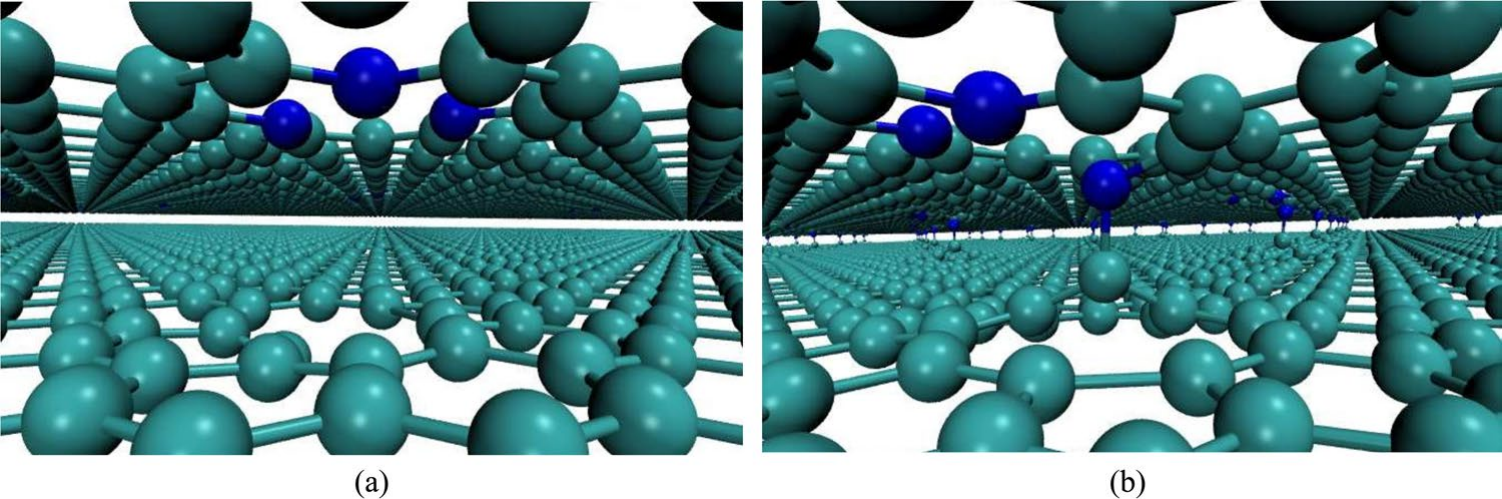
Defect complexes in bilayer graphene, trimerized pyridine combined with a single vacancy. (**a**) not-clipped and (**b**) clipped.

Having established that isolated defects in monolayer and bilayer graphene are not markedly different and thus are not expected to produce different XPS signatures, we investigate complexes of defects in the bilayer. Specifically, complexes in which defects are in adjacent layers. The proximity of two defects will not only induce structural distortions through electrostatic interaction, but also due to the presence of frustrated bonds, that can lead to significant reconstructions. In some cases, the interaction of defects in opposite layers of the bilayer can lead to the formation of covalent bonds between the layers. This phenomenon is called clipping^33^, and is illustrated in Fig. 5. In Table 1 we show the formation energy of the defect complexes (*E*_*f*_) calculated in the same supercell, and the difference of this formation energy relative to the sum of the formation energies of the defective monolayers that make up the defect complex (Δ*E*_*f*_). This difference in energy gives an idea of the interaction energy between the defective monolayers when combined. We also indicate whether the interlayer covalent bond remained stable during the optimization of the atomic positions in our simulations of these complexes and what are the formation energies when interlayer bonds do remain stable. As discussed previously for monolayer graphene and the bilayer with one isolated defect, we tested the effect of optB86b + vdW-DF functional in these results, as shown in Tables S2 and S3 and Fig. S4 in Supplemental Information. Unlike what happened for the isolated defects in the monolayer and the bilayer, the formation energy of defect complexes in the bilayer evaluated with optB86b + vdW-DF functional shows a clear rigid shift for all cases tested. Since we are interested in relative analyses among these systems, the rigid shift obtained through optB86b + vdW-DF functional is of little consequence for the rest of our discussion.

**Table 3:** Calculated binding energy for 1s core electrons of Nitrogen defects in bilayer graphene, two defective layers combined, AB stacked.

Defect	Clipped?	Position	N *E*_*CLS*_^1*s*^ (eV)
Trim. pyri. + SV	no	Pyri. N	4.5
	yes	Pyri. N*	2.3
		Pyri. N	4.4
Trim. pyrr. + SV	no	Pyrr. N	5.3
		Pyri. N	4.9
	yes	Pyrr. N*	2.2
		Pyri. N	4.5
Trim. pyrr. + subst.	no	Subst. N	1.4
		Pyrr. N	5.2
		Pyri. N	4.9

*Indicates the atom which forms an interlayer bond.

As shown in Table 1, the interaction between defective graphene layers in all cases studied lowered the energy of the system relative to the isolated defective monolayers. Our experimental analyses of the bilayer showed marked differences of the XPS as the content of nitrogen increased in the chamber where the samples were. This type of interaction between the layers mediated by defects can be the cause of this evolution of the XPS shown in Fig. 2, since this technique is sensitive to changes in the chemical environment where atomic species are located (*i.e*. different bonding arrangements).

We simulated the XPS experiment in our defective graphene bilayers computing the electron core level shift for different configurations of the defects. The core level shifts are not only related to the orbital of the electron within the nucleus of an atom but also to how this atom bonds with its neighbors. The difference in the bonding structure of an atom results in different binding energies for core electrons in the same atom. This energy difference is usually enough to distinguish between a nitrogen in the substitutional defect, Fig. 4b, and a nitrogen in a pyridine defect, Fig. 4f, for example^12^.

We computed the core eigenvalues using the final state approximation within VASP^55^. In this method, we compute the energy difference of a system in its ground state with the same system with one electron removed from the core of a specific atom. In the final state approximation, the electrons are allowed to relax, screening the localized core hole. This energy difference gives the binding energy of the respective core electron. Absolute energy values are not meaningful, but the core level shift, that is, the difference of the binding energy of the same core electron in different chemical environments (e.g. on a surface vs. in the bulk of a material) is accurate to within 50 meV^55^. Our referential is the core eigenvalue of one 1 *s* electron from substitutional nitrogen on monolayer graphene, see Fig. 4b. We use Eq. (1) to compute the core level shift (CLS) from this same referential for all cases.

1 $${E}_{CLS}={\varepsilon }_{c}^{mat}-{\varepsilon }_{1s}^{subst\,N}$$

where *E*_*CLS*_ is the energy of the core level shift, ${\varepsilon }_{c}^{mat}$ is the energy of the core level *c* in the configuration of interest and ${\varepsilon }_{1s}^{substN}$ is the core energy of one 1 *s* electron from substitutional nitrogen on monolayer graphene, this energy is −406.4 eV.

When considering the combination of a monolayer containing a nitrogen defect and a pristine monolayer, we observed an almost rigid translation in the core level shift, as shown in Table 2. These defects yield changes of CLS of about 1.5 eV. This is about the full width at half maximum in the experimental analysis, Fig. 3. In principle, the difference between the defects binding energies could be distinguished in an XPS experiment. For the single vacancy defect the energy shift is much larger, almost 4.9 eV. This is explained by the carbon atom with a dandling bond that was on the plane (monolayer), and that when combined with a pristine monolayer presents a distortion to the interlayer region, Fig. S5 in Supporting Information, also resulting in change of the formation energy, Table S2 in Supplemental Information. We note however that single vacancies are very unstable in graphene, monolayer or bilayer. Therefore, they are not expected to appear in our XPS.

When defects in adjacent layers interact, a significant difference between clipped and not-clipped structures can be seen (Table 3). Considering a trimerized pyridine defect in one layer and a single vacancy in the adjacent layer, when no interlayer bond is formed, all pyridinic positions yielded similar results. On the other hand, when considering the structure where one of the pyridinic nitrogens bonds covalently with the dangling carbon of a single vacancy in an adjacent layer, this nitrogen showed a difference of −2.2 eV in the CLS energy relative to the other two (see Table 3).

**Figure 6:**
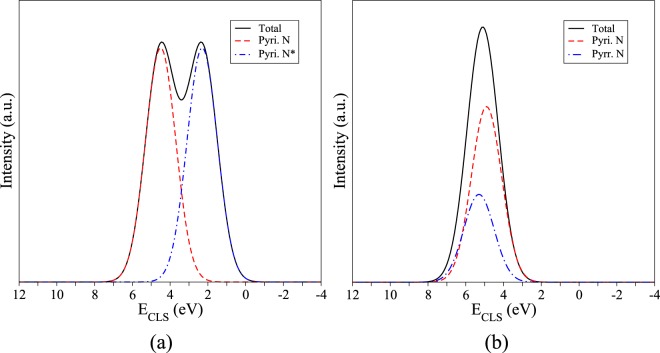
Simulated XPS pattern computed with Table 3 CLS. (**a**) Expected XPS pattern for clipped trimerized pyridine and single vacancy, similar to one observed in Fig. 3 for NG2 and NG3 samples; (**b**) Trimerized pyrrole and single vacancy showing that although present the pyrrole defect peak would be less significant that the peak observed for the other two pyridinic positions within the defect (see Fig. 4f) and due to its energy proximity it would be difficult to observed it experimentally.

When a pyrrole defect occurs in the geometry shown in Fig. 4f, two pyridinic nitrogens nearby are needed to stabilize the defect complex. In Table 3 we see that the core level shift for this trimerized pyrrole nitrogen combined with a single vacancy, clipped and not-clipped changed from 5.3 eV to 2.2 eV, a difference of −2.3 eV. This 5.3 eV energy shift is identical to the one obtained for the covalently bound nitrogen in the trimerized pyridine complex, discussed above, therefore these two defects may not be distinguishable in the XPS. We note that pyridine defects are expected to be orders of magnitude more frequent in our systems due to the much lower formation energy relative to the pyrrole (see Table S1 in Supplemental Information). These results are depicted in Fig. 6, where (a) shows the case where a clipped structure of pyridine defects results in different CLS energies for the same defects, due to one of the defects interaction with the adjacent layer, and (b) shows the case where a pyrrolic nitrogen and a pyridinic nitrogen are “obtained” in the same sample, the difference in concentration would yield a much higher peak for pyridinic nitrogen and since the CLS for both defects are near each other, a single peak would be seen.

As stated in the Experimental Results and Discussion, the analyses of the XPS survey (Supp. Info. Fig. S2) yields nitrogen concentrations of 2.0 at.%, 4.2 at.% and 4.2 at.% for samples NG1, NG2, and NG3, respectively. The saturation of nitrogen incorporation in sample NG3 relative to NG2 despite the higher flux is likely a consequence of the complete passivation of vacancies in the samples. As discussed above, the PECVD induces quite large concentrations of vacancies in the bilayer graphene, as indicated by the large D band in the Raman spectra show in Fig. 1. The incorporation of nitrogen on these defects is extremely favorable, given the very low formation energies indicated in Table S1 in Supplemental Information but without the presence of vacancies the incorporation of nitrogen in graphene is not favorable. The large concentration of vacancies due to the PECVD is also the reason for the high occurrence of pyridinic defects in our samples, since these defects require the presence of complexes of two to four vacancies in neighboring sites (see Fig. 4). Despite the concentration of nitrogen incorporated in samples NG2 and NG3 being numerically equivalent, the XPS curves indicate a qualitative change in the nature of the defect complexes between these samples. The area of the split off XPS peak, that our simulations have associated to nitrogen atoms forming interlayer bonds, indicate an increase in their atomic concentrations from 0.5 at.% to 2.0 at.% and to 2.7 at.% from samples NG1 to NG2 and NG3, respectively. The reason for this qualitative change between samples NG2 to NG3 despite the similar overall nitrogen content is likely to be some dynamical effect not captured by our static DFT simulations and further analyses would be necessary to clarify such mechanism. The occurrence of interlayer bonds and their connection to the split off peak seen in the XPS signal, however, have been conclusively demonstrated by our simulations.

**Figure 7:**
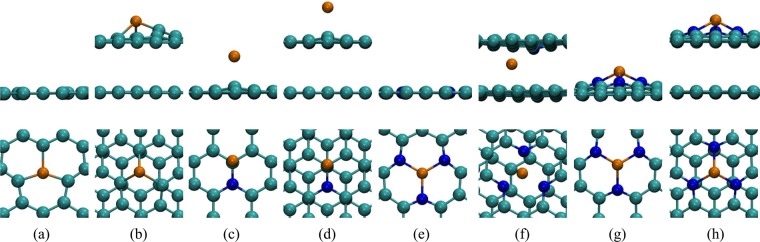
Copper defect complexes in graphene: (**a**) substitutional Cu in monolayer and (**b**) bilayer graphene, (**c**) substitutional N with an adsorbed Cu in monolayer and (**d**) bilayer graphene, (**e**) trimerized pyridine N with a Cu adatom into the vacancy of monolayer and (**f**) bilayer graphene, and (**g**) trimerized pyridine N with Cu above the vacancy on monolayer and (**h**) bilayer graphene.

Finally, we also investigated defect complexes involving copper impurities. Since we used Cu substrate to grow and analyze our samples, there is a possibility that this impurity could be responsible for some of the features observed in our experimental XPS analyses. As a matter of fact, other authors have proposed this interaction of Cu with N defects as the cause of the splitting in the XPS peak that we have also observed^49^. However, as mentioned above, copper nitrate is not stable at our experimental conditions (760 °C temperature synthesis). To further verify this conclusion, we analyzed cooper defects on monolayer and bilayer graphene. In Fig. 7 we show the complexes simulated: substitutional Cu, substitutional N with an adsorbed Cu atom, trimerized pyridine N with a Cu into the vacancy and trimerized pyridine N with a Cu above the vacancy. Once the formation energy of the trimerized pyridine N defects are lower than the trimerized pyrrole one (Tables S1 and S2 in Supplemental Information) and thus the concentration of the pyridine complex defect is predicted to be much higher than the pyrrole, we considered only the defects of copper complexes formed with trimerized pyridine structure.

The results of formation energies and CLS regarding the copper-related defect complexes described above can be found in Table 4. We notice that the formation energies of the considered defects tend to be lower in bilayer graphene, except for substitutional N with Cu adsorbed. This indicates that the presence of the second graphene layer favours a stabilization of defects containing Cu atoms. More importantly, however, is to note that the formation energies of cooper-related defect complexes are higher than that of most N-related defects. Therefore, the concentration of defects with Cu atoms in the graphene will be fairly low. This is in agreement with the experimental reports which have indicated that the copper substrate remains inert during the growth of the graphene onto its surface^26,39,47,48,56^.

**Table 4:** Formation energies *E*_*f*_ of the copper-related defect complexes and the calculated CLS for N1 s core electrons in monolayer and bilayer graphene.

Defects	*E*_*f*_^*LDA*^ (eV)		N *E*_*CLS*_^1*s*^ (eV)	
	Mono.	Bi.	Mono.	Bi.
Subst. Cu	9.44	7.85	—	—
Subst. N + Cu adsorbed	3.87	4.92	29.6	28.2
Trim. pyri. + Cu into SV	3.43	1.73	27.7	25.6
Trim. pyri. + Cu above SV	2.66	2.57	27.5	25.9

These CLS results were obtained considering the N1s core eigenvalues.

Considering the CLS of nitrogen atoms in the copper-related defect complexes, from monolayer to bilayer graphene, we verified a rigid shift of at most 2 eV among the results of all the analyzed defects. Moreover, the nitrogen CLS energies of the copper-related defect complexes are about 20 eV distant from those values obtained for the nitrogen defects (see Table 2). This result refutes any possibility that the presence of N-Cu interactions could be responsible for the splitting in the XPS signal observed by us and other authors. Therefore, we conclude that as indicated above, the splitting in the XPS signal of N-related defects in bilayer graphene is associated to energy level shifts of core electrons on the nitrogen atom that bridges the two layers of the material through a covalent bond.

## Conclusions

We described a MW-PECVD process that enables the growth of nitrogen-doped bilayer graphene films on Cu foil in a fast and reproducible way, producing films with large area (5 mm × 5 mm) and high crystallinity. The plasma/metal coupling causes localized, rapid heating of the foil, leading to a fast synthesis (2.5 min). This localization also reduces the post-growth cooling time (10 min). The combination of hydrogen, nitrogen and methane plasma serves to remove the copper native oxide layer as the synthesis occur, minimizing the number of steps taken to obtain a stable graphene sheet. It was also shown that the change in N_2_:CH_4_ proportion allowed the control of the concentration of dopants in the final samples. XPS analyses identified a splitting in the signal associated to nitrogen defects as their concentration increased. We found no reports of a similar phenomenon in the literature for nitrogen doped graphene monolayers.

We performed state-of-the-art, parameter-free simulations based on the density functional theory (DFT) to verify the cause of this splitting of the XPS signal. Using the local density approximation as well as implementations of the DFT that explicitly include van der waals interactions (optB86b + vdW-DF) we obtained the same trend for the formation energy of several monolayer graphene defects, bilayer graphene defects and defect complexes. When defects are close enough in adjacent layers, they can induce the formation of interlayer covalent bonds, a phenomenon known as “clipping”. Calculations of the shifts in core level energy of these interacting defect complexes suggest that the features observed in the high resolution N1s XPS spectrum of doped samples (NG2 and NG3) are related to the clipping of defect complexes in bilayer graphene. The formation of interlayer bonds changes the chemical environment around the impurity atom, affecting the core electron binding energy detected by the XPS measurement. Our simulations show that interaction between defect complexes are highly dependent on experimental conditions and can be tuned by controlling the ratio of N_2_ during synthesis. These nitrogen related defects must be further studied, but are expected to alter mechanical and electronic properties of bilayer graphene in a markedly different way from monolayer graphene. These defects are likely to be present in other types of few layer 2D films and heterostructures and we indicate a route to identify their occurrence through XPS characterization and computer simulations. Moreover, their are likely to play a role in the new field of *twistronics* that needs to be further investigated. The suppression or incorporation of these defects in 2D few layer structures can be another parameter to tune in order to adjust their properties for specific applications.

## Methods

### Experimental methods

Graphene films were grown in an in-house built microwave plasma-enhanced chemical vapor deposition (MW-PECVD) system, which configuration is described in details elsewhere^57^. In this system, the sample is mounted onto the top of a quartz tube cap inside the plasma chamber, and the bottom surface of the sample can be viewed by an optical pyrometer positioned outside the chamber. Copper films with dimensions of 5 × 5 × 0.025 mm^3^ were used as substrates. First the MW-PECVD chamber was pumped down to 15 Torr, followed by an argon purge, in which the system reached a pressure of 30 Torr. The synthesis was then started by plasma activation. To start the plasma, the microwave power was adjusted to a value of 500 W, resulting in a voltage of 85 V, and generating a locally confined plasma of ~5 cm diameter around the sample. The plasma parameters resulted in the heating of the substrate to 760 °C, which remained constant throughout the synthesis. After the plasma activation, the argon flow was interrupted and the 10 standard-cubic centimeters-per-minute (s.c.c.m.) of room temperature hydrogen gas was added. Keeping the hydrogen flow constant, several flow ratios of methane and nitrogen gases were added into the chamber, as described in Table 5, for pristine (PG) and nitrogen doped (NG) graphene growing. The gas mixture was uniformly dispersed in the chamber via an annular gas feed system. The pressure was kept at 43 Torr during 2.5 mm of synthesis. Finally, plasma was extinguished, hydrogen, methane and nitrogen flows were ceased and the chamber was filled with argon for the substrate cooling up to room temperature, being the average cooling time 10 mm. With this, pristine and doped bilayer structures were obtained. The synthesis of monolayer structures was not achieved with the described system.

**Table 5:** Parameters applied for the synthesis of pristine (PG) and nitrogen doped (NG) graphene.

Sample	N_2_:CH_4_ Flux ratio (s.c.c.m.)	Temperature (°C)	Synthesis time (min)	Pressure (Torr)	Voltage (V)
PG	1:1	760	2.5	43	85
NG1	2:1	760	2.5	43	85
NG2	3:1	760	2.5	43	85
NG3	5:1	760	2.5	43	85

All samples were synthesized at a constant flow of 10 s.c.c.m. hydrogen.

For Raman spectroscopy, graphene films were transferred onto a 300 nm thick SiO_2_ layer on Si by PMMA transfer method in order to obtain a spectra without copper background reflectance interference^6,58^. Raman spectroscopy was performed on a confocal micro-Raman spectrometer (Witec Alpha 300R), equipped with a 532 nm laser source (power at sample below 0.1), 100× objective and charge coupled device (CCD) detector. The system generated a laser spot size of 300 mm (diameter) and spectral resolution of 3 cm^−1^. The wavenumber calibration was based on standard values for crystalline silicon band checked at 532 cm^−1^. Measurements were carried out at room temperature and peaks were fitted using a Lorentzian function.

Film composition was investigated by X-ray photoelectron spectroscopy (XPS) using a UNI-SPECS UHV spectrometer with monochromatic Al *K*_*α*_ radiation (*hν* = 1486.6 eV) and pass energy of 10 eV, operating at base pressure of 3e-9 Torr. Background was subtracted according to Shirley model, and peak fitting was performed with a product of Gaussian and Lorentzian shapes. Initial quantification was based on Scofield sensitivity factors^59^ and a model of uniform elemental distribution. The chemical composition of the samples (PG, NG1, NG2 and NG3) were estimated from survey XPS spectra as shown in Fig. S2 for the sample NG3 before the transference to silicon substrate, where the peak areas were determined after background subtraction using the Shirley’s method.

### Simulation details

All simulations were performed with *ab initio* total energy calculations based on the density functional theory (DFT)^60,61^ with the Vienna *Ab Initio* Simulation Package (VASP)^62,63^ using the local density approximation (LDA)^64^. Results obtained by this approximation were compared with results using van der Waals corrections through optB86b + vdW-DF exchange and correlation functional^50–54^. For the Brillouin zone sampling, an optimized Γ-centered 1 × 1 × 1 mesh was used for geometry relaxations and Γ-centered 2 × 2 × 1 for electronic properties. The atomic positions in all structures were relaxed using the CG algorithm until residual forces were below 0.025 eVÅ^−1^. Pristine monolayer and bilayer graphene supercells considered have 160 and 320 atoms, respectively. We used 20 Å of vacuum in the *z* direction to prevent spurious interaction between our simulated supercells and their images given that periodic boundary conditions (pbc) are used. The projector augmented-wave method (PAW)^65^ was used and the kinetic energy cutoff for the plane wave expansion was optimized to 560 eV.

## Supplementary information

Characterization of nitrogen doped graphene bilayers synthesized by fast, low temperature microwave plasma-enhanced chemical vapor deposition

## Data Availability

All data generated or analysed during this study are included in this published article (and its Supplementary Information Files).
