# Publisher Correction: Characterization of nitrogen doped graphene bilayers synthesized by fast, low temperature microwave plasma-enhanced chemical vapour deposition

**DOI:** 10.1038/s41598-019-54234-7

**Published:** 2019-11-21

**Authors:** C. R. S. V. Boas, B. Focassio, E. Marinho, D. G. Larrude, M. C . Salvadori, C. Rocha Leão, D. J. dos Santos

**Affiliations:** 1 Federal University of ABC, Centro de Engenharia, Modelagem e Ciências Sociais Aplicadas, Santo André, 09210-580 Brazil; 2 University of Twente, Industrial Focus Group XUV Optics, MESA+ Institute for Nanotechnology, Enschede, 7522 NH The Netherlands; 3 Graphene and Nano-materials Research Center - MackGraphe, Mackenzie Presbyterian University, São Paulo, 01302-907 Brazil; 4 Physics Institute, University of São Paulo, São Paulo, 05508-090 Brazil

Correction to: *Scientific Reports* 10.1038/s41598-019-49900-9, published online 23 September 2019

The original version of this Article contained an error in the title of the paper, where the word “graphene” was incorrectly given as “grapheme”. This has now been corrected in the PDF and HTML versions of the Article.

