# Supplementary material for: Characterization of nitrogen doped graphene bilayers synthesized by fast, low temperature microwave plasma-enhanced chemical vapour deposition
Source: Sci Rep. 2019 Sep 23;9:13715. doi: 10.1038/s41598-019-49900-9 (PMC6757055; doi:10.1038/s41598-019-49900-9)
Supplement: Supplementary file 1 — Characterization of nitrogen doped graphene bilayers synthesized by fast, low temperature microwave plasma-enhanced chemical vapor deposition [file 41598_2019_49900_MOESM1_ESM.pdf]

Characterization of nitrogen doped graphene bilayers synthesized by fast, low temperature microwave plasma-enhanced chemical vapor deposition

C. R. S. V. Boas[1,2,+], B. Focassio[1,+], E. Marinho Jr[1], D. G. Larrude[3], M. C. Salvadori[4], C. Rocha Leão[1,*], and D. J. Santos[1,*]

[1]Federal University of ABC, Centro de Engenharia, Modelagem e Ciências Sociais Aplicadas, Santo André, 09210-580, Brazil
[2]University of Twente, Industrial Focus Group XUV Optics, MESA Institute for Nanotechnology, Enschede, 7522 NH, The Netherlands
[3]Graphene and Nano-materials Research Center - MackGraphe, Mackenzie Presbyterian University, São Paulo, 01302-907, Brazil
[4]Physics Institute, University of São Paulo, São Paulo, 05508-090, Brazil
[*]cedric.rocha@ufabc.edu.br
[*]demetrio.santos@ufabc.edu.br
[+]these authors contributed equally to this work

1 Suplementary Information

1.1 Experiment

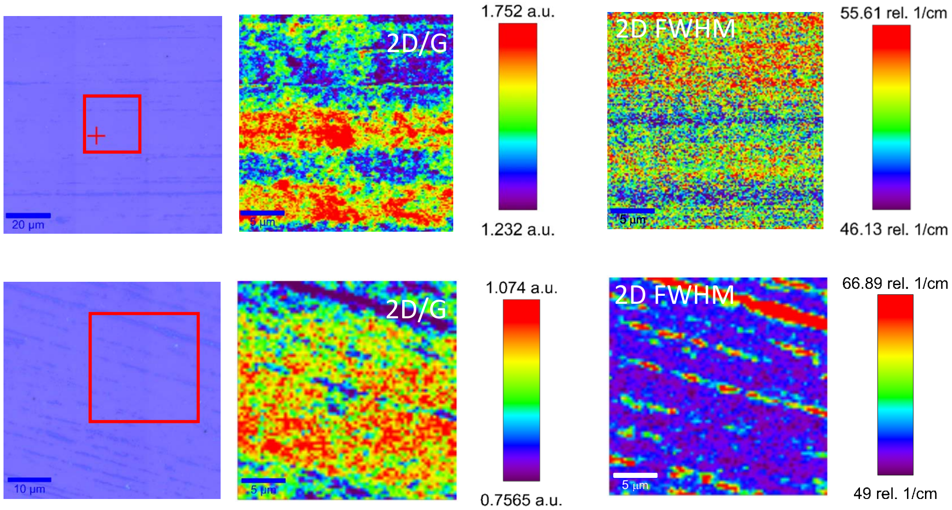

Figure 1. Raman mapping of of PG (top) and NG1 (bottom) samples with optical image, 2D FWHM and I_{2D}/I_G intensity ratio. The mapped areas are highlighted by red squares in the optical image. A mean FWHM of PG 50.8 cm^{-1} and 51.6 cm^{-1} for NG1, and a mean I_{2D}/I_G of 1.1 and 0.9 is shown respectively for each sample.

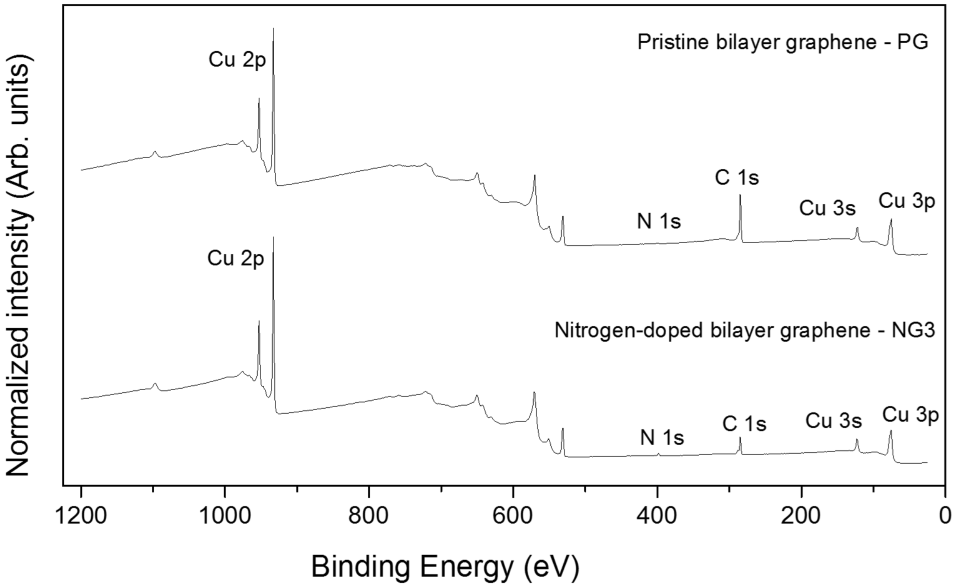

Figure 2. XPS survey spectra of PG and NG3 samples. The core level N 1s, C 1s, Cu 2p, Cu 3p and Cu 3s peaks found in the spectrum of PG and NG3 confirm the presence of the constituent elements in the samples.

1.2 Theory

In order to have a consistent analyses for the bilayer graphene, we started with several calculations for the monolayer system. Also, we validated our results using pseudopotentials in which van der Waals interactions are explicitly included.

We simulated the monolayer graphene structure first using the local density approximation (LDA), as described in the theoretical methodology. We obtained a C-C bond length of 1.41 Å, an error of 0.7% , in agreement with previous theoretical results[1–3]. In thermodynamic equilibrium, the proportion of defects in a material varies exponentially with the energy needed to form each defect (E_f), via a Boltzmann factor $F = C_0 \exp\left(-E_f/k_B T\right)$ where C_0 is the concentration of available incorporation sites per volume or area for the defect. However, experimental conditions can result in systems far from thermodynamic equilibrium, and if the energy barriers are high, the relaxation time towards this equilibrium can be very long. The formation energy can be calculated with the equation (1).

$$E_f[X] = E_{tot}[X] - E_{tot}[pristine] - \sum n_i \mu_i \tag{1}$$

where $E_{tot}[X]$ is the total energy of the supercell containing the defect X, $E_{tot}[pristine]$ is the total energy of the pristine supercell of the material considered, n_i is the number of atoms of species i added (> 0) or removed (< 0) to form the defect and μ_i is the atomic chemical potential of species i in its bulk phase. i.e., graphite for carbon, N_2 for nitrogen and FCC for copper. In table 1 we show the formation energy of defects in graphene monolayer obtained using our methodology.

The formation energy of the single vacancy (SV) is high[4,5], as a consequence of the dangling bonds left by the removal of a carbon atom and the distortion introduced by the formation of a pentagon involving the remaining carbon atoms[4,6,7]. Also, the formation energy of the substitutional nitrogen defect is the lowest of all tested defects, since it does not induce significant deformation of the neighboring bonds[8]. For the trimerized pyridine geometry (figure 4 e in the main article) the value of 2.54 eV is similar to previous reports[8]. This energy is lower than the formation energy of the SV, and this is attributed to the elimination of the carbon pentagon and stabilization of the three hexagons with nitrogens, reducing structural deformation, which does not occur for the other pyridine defects, figures 4 c and d in the main article) The pyrrole defect's, figure 4 f in the main text, high formation energy when compared to the pyridine is a result of the deformation caused by the formation of a pentagon containing one dangling nitrogen bond.

Understanding the electronic nature of these defects is important to predict their tendency to combine in complexes, specially in bilayer graphene, when defects in adjacent layers might attract each other, forming complexes. The simulation of charged defects with periodic boundary conditions (pbc), however, presents a significant challenge in extended systems that contain a vacuum layer, such as surfaces, 2D and 1D materials. The inclusion of a jellium field solves the problem of

the diverging electrostatic charge. However, the spurious interaction among the supercell and its images remains a sensitive topic. Unlike for bulk systems, in which enlarging the size of the supercell permits estimating when this spurious electrostatic interaction is minimized to acceptable levels, the simulation of systems that are extended in one, two or three dimensions is far more complicated. Enlarging the supercell with a vacuum reduces the screening of the background opposing field. Moreover, Makov-Payne corrections[9] cannot be easily estimated since the dielectric screening is anysotropic[10].

Therefore, here we resort to an analysis of the electronic density of states (DOS) relative to the Fermi level to verify if the defect complexes are positively or negatively charged[11]. Figure 3 shows the pristine, substitutional nitrogen, trimerized pyridine and pyrrole defects DOS. We indicate in those images 3 the point where the Fermi level crosses the DOS curve. If this point occurs at an energy above the pseudogap of graphene, there are populated levels at the conduction band, indicating that the defect complex is negatively charged 3(b). Similarly, if the Fermi level crosses the DOS curve at an energy lower than that of the pseudogap of graphene, there are empty levels at the valence band, indicating the defect complex is positively charged,3(c) - 3(d).

Defect	E_f^{LDA} (eV)	Charge state
Single vacancy	8.15	+
Substitutional	0.25	-
Monomerized pyridine	4.95	+
Dimerized pyridine	4.27	+
Trimerized pyridine	2.54	-
Trimerized pyrrole	4.68	+

Table 1. Formation energy using LDA exchange and correlation functional and charge state of monolayer graphene defects studied.

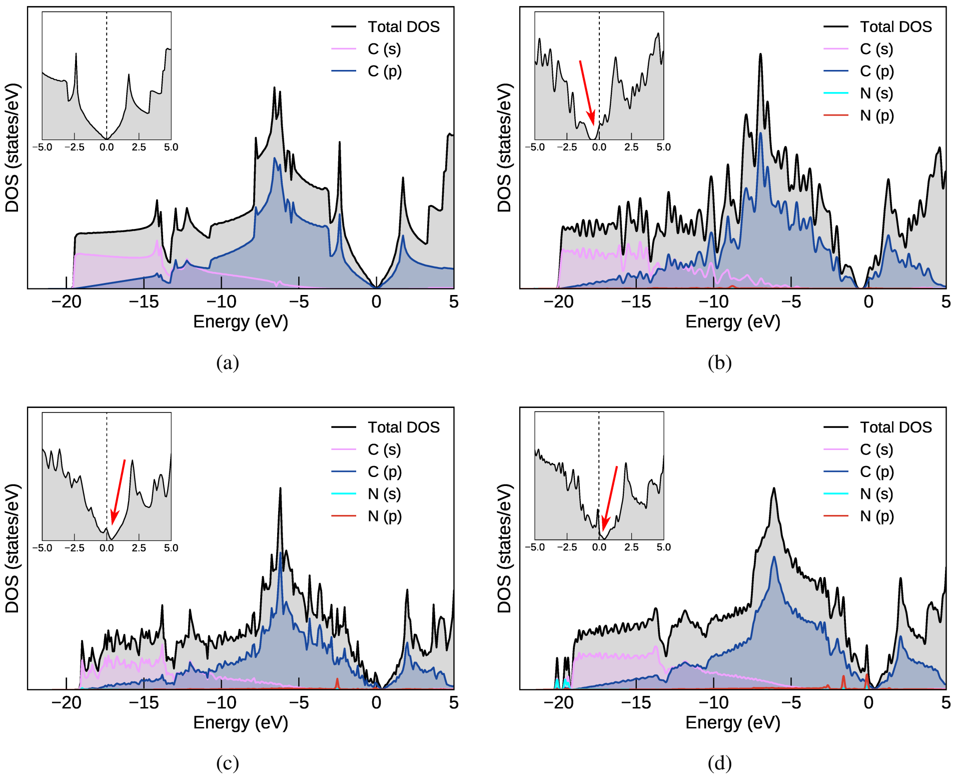

Figure 3. Electronic density of states of monolayer graphene obtained with LDA. (a) pristine; (b) substitutional nitrogen; (c) trimerized pyridine and (d) trimerized pyrrole defect. The inserts shows the region around the Fermi level magnified, The valence band maximum is shifted to the Fermi level (zero of x-axis). DOS results were carefully checked for consistency comparing with spin polarized calculations for selected systems.

We simulated bilayer graphene in the AA and AB stacking using the local density approximation (LDA) for the exchange-correlation term in the Kohn-Sham equations, as done to simulate monolayer. The total energy of AB stacked bilayer graphene is 5 meV per atom lower than that of AA stacked and the interlayer distances of AB and AA stackings are 3.33 Å and 3.61 Å, respectively. Both stackings still present C–C bond length of 1.41 Å. Our results are in good agreement with other reports[1–3]. We found that the binding energy between the layers pristine AB bilayer graphene is −11 meV per carbon atom, in agreement with the previous results using LDA[12,13].

Table 2 shows the formation energy of the resulting defects, calculated with the LDA approximation. We also show the formation energy difference of these defects relative to the monolayer, indicated in table 1 in supplemental material. In absolute values, formation energies vary around 0.13 eV, except for the vacancy in which the variation is larger. This is not so meaningful since the formation energy of this defect is almost two times larger than the next least frequent defect, trimerized pyrrole. The trimerized pyridine defect was the only one in which the formation energy became slightly larger in the bilayer than in the monolayer (5.5%). Despite these minor peculiarities, these results show that there are no significant changes in the proportion among the different defects isolated in the monolayer relative to the bilayer graphene when one of the layers is free from defects (pristine).

Defect	E_f^{LDA} (eV)	ΔE_f (eV)
Single vacancy	7.89	0.26
Substitutional	0.12	0.13
Monomerized pyridine	4.83	0.11
Trimerized pyridine	2.68	0.14
Trimerized pyrrole	4.56	0.12

Table 2. Formation energy of defects in AB bilayer graphene obtained with the LDA approximation, ΔE_f values considering the same defect in the monolayer supercell. Combination between a defective and pristine layer.

LDA is known to reproduce structural parameters in agreement with experimental data for several systems in which van der Waals (vdW) interactions play an important role. Often this agreement is assigned to fortuitous error cancellation in this functional. Other properties of the system, specially when defects are taken into account, could in principle be poorly described by LDA alone, regardless of the accordance in reproducing experimental data for the pristine material. Therefore, in order to verify our results for defects in bilayer graphene, we used optB86b+vdW-DF exchange and correlation functional[14–18] which aims to accurately describe van der Waals forces in DFT simulations. The interlayer distances of AB and AA stackings considering vdW forces remained nearly the same as those obtained with LDA 3.34 Å and 3.54 Å. The binding changed markedly to −33 meV per atom, or three times more than predicted by LDA. We also simulated defects using optB86b+vdW-DF functional, and for consistency, we reproduced the analysis carried out in monolayer graphene as well. In figure 4 we compare results using these two approaches. Interestingly we only observe a rigid shift upwards in the formation energy of defects treated with optB86b+vdW-DF functional relative to LDA in the case of defect complexes in bilayer graphene, figure 4(c). These results are also reproduced in table 4.

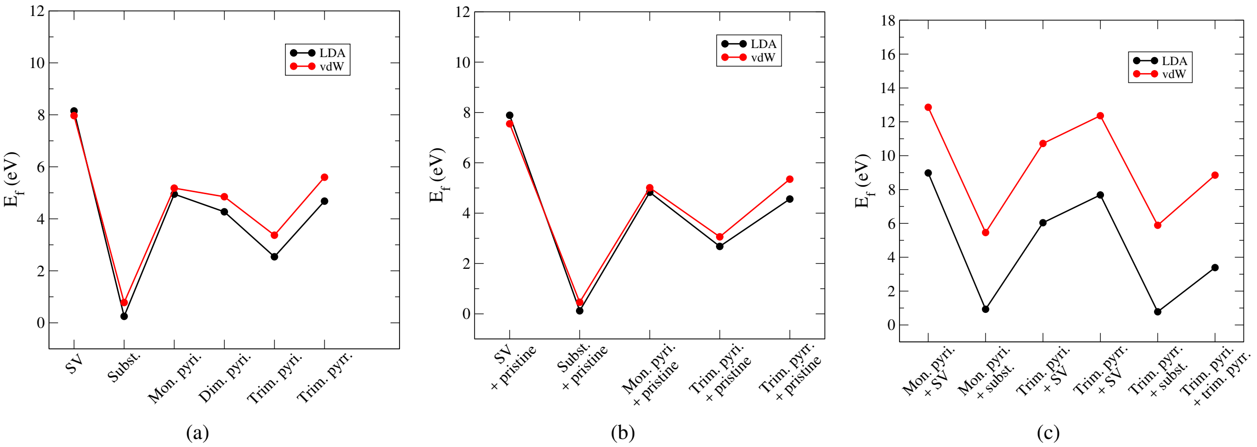

Figure 4. Formation energy of graphene defects studied, comparing LDA and vdW results showing a rigid energy shift. (a) Monolayer graphene defects, (b) Bilayer graphene defects obtained combining a defective and a pristine layer, (c) Bilayer graphene defect complexes.

Defect	Clipped ?	E_f^{vdW} (eV)	ΔE_f^{vdW} (eV)
Mon. pyri. + SV	no	12.78	0.07
Mon. pyri. + subst.	no	5.39	0.28
Trim. pyri. + SV	no	10.87	0.18
	yes	10.64	0.41
Trim. pyrr. + SV	no	13.16	0.12
	yes	12.28	1.00
Trim. pyrr. + subst.	no	5.82	0.28
Trim. pyrr. + Trim. pyri.	no	8.78	0.09

Table 3. Defect complex formation energy E_f using vdW corrections, ΔE_f is the difference between the bilayer formation energy of the bilayer structure formed by two defects E_f and the formation energy of each defect in its monolayer graphene structure, $\Delta E_f = E_{f,12} - (E_{f,1} + E_{f,2})$.

Finally, in figure 5 we show the interlayer bond that forms between the layers of graphene for some defect complexes. These bonds significantly change the XPS signal of the atoms involved, as discussed in the main text.

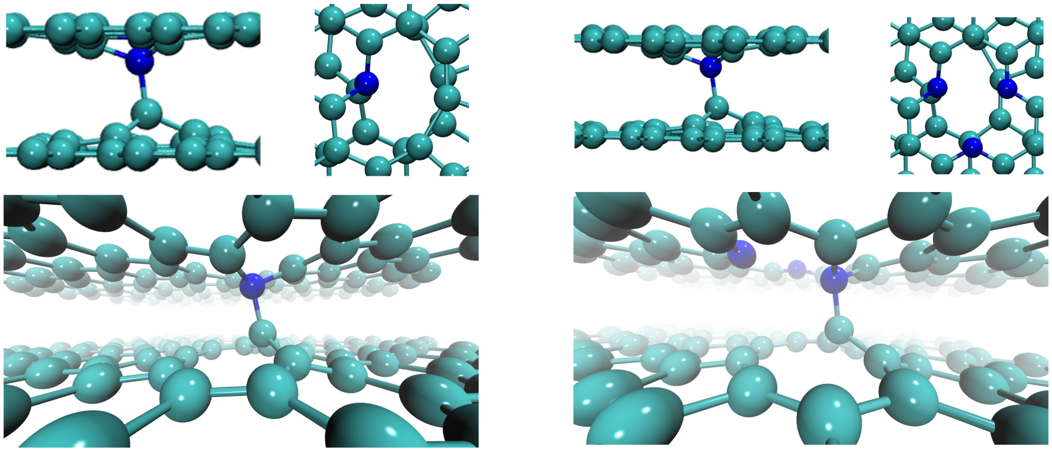

Figure 5. Interlayer bonds formed by the interaction of a monomerized piridin defect (a) and a trimerized pirrole defect (b) with a carbon vacancy in the adjacent layer.

References

1. Alam, M. S., Lin, J. & Saito, M. First-principles calculation of the interlayer distance of the two-layer graphene. *Jpn. J. Appl. Phys.* **50**, 080213 (2011).
2. Omata, Y., Yamagami, Y., Tadano, K., Miyake, T. & Saito, S. Nanotube nanoscience: A molecular-dynamics study. *Phys. E* **29**, 454 – 468 (2005).
3. Fujimoto, Y. & Saito, S. Electronic structures and stabilities of bilayer graphene doped with boron and nitrogen. *Surf. Sci.* **634**, 57–61 (2015).
4. Krasheninnikov, A., Lehtinen, P., Foster, A. & Nieminen, R. Bending the rules: Contrasting vacancy energetics and migration in graphite and carbon nanotubes. *Chem. Phys. Lett.* **418**, 132–136 (2006).
5. El-Barbary, A. A., Telling, R. H., Ewels, C. P., Heggie, M. I. & Briddon, P. R. Structure and energetics of the vacancy in graphite. *Phys. Rev. B* **68**, 144107 (2003).
6. Saito, M., Yamashita, K. & Oda, T. Magic numbers of graphene multivacancies. *Jpn. J. Appl. Phys.* **46**, L1185 (2007).
7. Amorim, R. G., Fazzio, A., Antonelli, A., Novaes, F. D. & da Silva, A. J. R. Divacancies in graphene and carbon nanotubes. *Nano Lett.* **7**, 2459–2462 (2007).
8. Fujimoto, Y. Formation, energetics, and electronic properties of graphene monolayer and bilayer doped with heteroatoms. *Adv. Condens. Matter Phys.* **2015** (2015).
9. Makov, G. & Payne, M. Periodic boundary conditions in ab initio calculations. *Phys. Rev. B* **51**, 4014 (1995).
10. Leão, C. R., Fazzio, A. & da Silva, A. J. Confinement and surface effects in b and p doping of silicon nanowires. *Nano Lett.* **8**, 1866–1871 (2008).
11. Liu, Y., Weinert, M. & Li, L. Determining charge state of graphene vacancy by noncontact atomic force microscopy and first-principles calculations. *Nanotechnology* **26**, 035702 (2014).
12. Björkman, T., Gulans, A., Krasheninnikov, A. V. & Nieminen, R. M. Are we van der waals ready? *J. Phys.: Condens. Matter* **24**, 424218 (2012).
13. Rêgo, C. R., Oliveira, L. N., Tereshchuk, P. & Da Silva, J. L. Comparative study of van der waals corrections to the bulk properties of graphite. *J. Phys.: Condens. Matter* **27**, 415502 (2015).
14. Dion, M., Rydberg, H., Schröder, E., Langreth, D. C. & Lundqvist, B. I. Van der waals density functional for general geometries. *Phys. Rev. Lett.* **92**, 246401 (2004).
15. Román-Pérez, G. & Soler, J. M. Efficient implementation of a van der waals density functional: Application to double-wall carbon nanotubes. *Phys. Rev. Lett.* **103**, 096102 (2009).

16. Klimeš, J., Bowler, D. R. & Michaelides, A. Chemical accuracy for the van der waals density functional. *J. Phys.: Condens. Matter* **22**, 022201 (2009).

17. Klimeš, J., Bowler, D. R. & Michaelides, A. van der waals density functionals applied to solids. *Phys. Rev. B* **83**, 195131 (2011).

18. Thonhauser, T. *et al.* van der waals density functional: Self-consistent potential and the nature of the van der waals bond. *Phys. Rev. B* **76**, 125112 (2007).
